# Longitudinal single-cell data informs deterministic modelling of inflammatory bowel disease

**DOI:** 10.1038/s41540-024-00395-9

**Published:** 2024-06-24

**Authors:** Christoph Kilian, Hanna Ulrich, Viktor A. Zouboulis, Paulina Sprezyna, Jasmin Schreiber, Tomer Landsberger, Maren Büttner, Moshe Biton, Eduardo J. Villablanca, Samuel Huber, Lorenz Adlung

**Affiliations:** 1 I. Department of Medicine, University Medical Center Hamburg-Eppendorf (UKE), D-20246 Hamburg, Germany; 2 Leibniz Institute for the Analysis of Biodiversity Change, D-20146 Hamburg, Germany; 3 Department of statistics and data science, Hebrew University of Jerusalem, Jerusalem, Israel; 4 Calico Life Sciences, LLC, South San Francisco, CA USA; 5 Department of Immunology and Regenerative Biology, Weizmann Institute of Science, Rehovot, Israel; 6 Division of Immunology and Allergy, Department of Medicine Solna, Karolinska Institutet and University Hospital, Stockholm, Sweden; 7 Center of Molecular Medicine, Karolinska Institutet, Stockholm, Sweden; 8 Hamburg Center for Translational Immunology (HCTI) and Center for Biomedical AI (bAIome), University Medical Center Hamburg-Eppendorf (UKE), D-20246 Hamburg, Germany

**Keywords:** Gastroenterology, Immunology, Computational biology and bioinformatics

## Abstract

Single-cell-based methods such as flow cytometry or single-cell mRNA sequencing (scRNA-seq) allow deep molecular and cellular profiling of immunological processes. Despite their high throughput, however, these measurements represent only a snapshot in time. Here, we explore how longitudinal single-cell-based datasets can be used for deterministic ordinary differential equation (ODE)-based modelling to mechanistically describe immune dynamics. We derived longitudinal changes in cell numbers of colonic cell types during inflammatory bowel disease (IBD) from flow cytometry and scRNA-seq data of murine colitis using ODE-based models. Our mathematical model generalised well across different protocols and experimental techniques, and we hypothesised that the estimated model parameters reflect biological processes. We validated this prediction of cellular turnover rates with KI-67 staining and with gene expression information from the scRNA-seq data not used for model fitting. Finally, we tested the translational relevance of the mathematical model by deconvolution of longitudinal bulk mRNA-sequencing data from a cohort of human IBD patients treated with olamkicept. We found that neutrophil depletion may contribute to IBD patients entering remission. The predictive power of IBD deterministic modelling highlights its potential to advance our understanding of immune dynamics in health and disease.

## Introduction

The advent of single-cell mRNA-sequencing (scRNA-seq) technologies has elucidated various facets of the immune system, ultimately facilitating our understanding of disease aetiology as a dynamic process^1^. Despite the increasing availability of high-throughput data, its implementation in predictive dynamical models is hampered by insufficient temporal resolution. As a single measurement can capture multiple cellular states along a temporal continuum, many computational approaches seek to exploit this to reconstruct developmental trajectories^2^. Fate mapping and barcoding provide additional information to trace immune cellular histories^3^. Where omics datasets do have temporal resolution, statistical modelling frameworks are available to explore and analyse them^4–6^. However, when looking for associations between measured traits and experimental conditions, such statistical models treat time as a confounding factor.

In general, scRNA-seq technologies are suitable for the identification and in-depth molecular characterization of immune cell types and cellular states^7,8^. Transcriptional profiles reveal cellular heterogeneity, reflecting distinct functional states with potential clinical relevance^9^. The kinetics of these cell states can be used to dynamically model disease progression. While statistical models typically use data to infer disease trends and associations, dynamic models use deterministic equations to explicitly represent the underlying biological processes and predict disease dynamics based on established principles. Mathematical models in these cases describe changes in the abundance of cell populations over time based on a set of coupled ordinary differential equations (ODEs). To describe experimental data, parameters must be estimated that represent rate constants of biological processes such as proliferation, differentiation, or death. ODE models are useful for simulating the dynamics of disease and discovering the principles governing in the progression of pathologies^10^.

Here, we used inflammatory bowel disease (IBD) as a paradigm for a dynamic immunological disease. IBD is characterised by relapsing-remitting inflammation of the gastrointestinal tract, associated with cramping, diarrhoea, and weight loss. The two most common forms of IBD in humans are Crohn’s disease (CD)^11^ and ulcerative colitis (UC)^12^. Mouse models of the latter include T-cell transfer colitis and dextran sodium sulfate (DSS) colitis^13^. Omics technologies have great potential to inform the treatment of IBD patients through personalized medicine^14,15^. In this work, we explored single-cell-based data from colitis in mice for deterministic modelling of IBD cell population dynamics. We aimed to create a generalizable ODE-based model indicative of biological processes during active IBD and entering remission. We propose that such a systems biology approach can contribute to a better mechanistic understanding of processes during immune-mediated inflammatory diseases.

## Results

### Longitudinal flow cytometry data reveals population dynamics during murine colitis

**Fig. 1:**
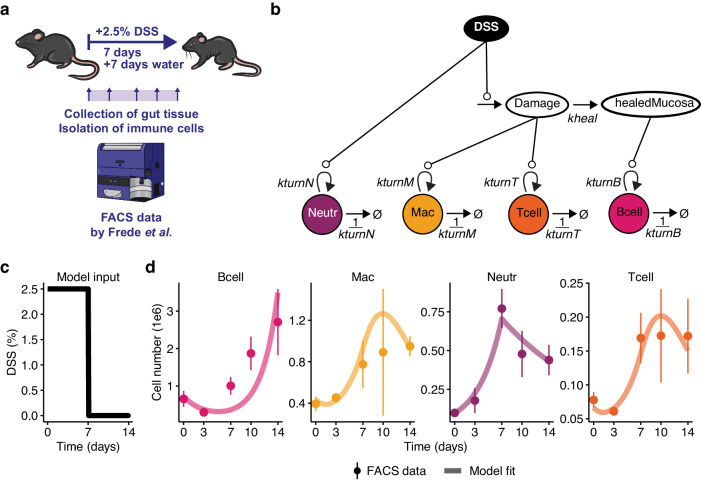
Schemes and model fit for FACS data. **a** Scheme of the FACS experiment published by Frede et al. **b** Schematic representation of the core mathematical model. **c** DSS treatment simulated as model input. **d** Experimental FACS data and simulations of the fitted model. Points represent mean values; error bars indicate standard deviations of experimental data from *N* = 3 mice per time point. Solid lines represent model simulations of the best fit.

As a first step to studying population dynamics during murine colitis with single-cell-based approaches, we chose longitudinal flow cytometry data of colonic immune cells, which we have previously published^16^. In this protocol, mice were treated for seven days with 2.5% DSS in the drinking water followed by seven days of water (Fig. 1a). At days 0, 3, 7, 10, and 14 of the experiment, each three mice were sacrificed, and total number of major immune cell types was determined by fluorescence-activated cell sorting (FACS). As a proof of concept, we focused on B cells, macrophages (Mac), neutrophils (Neutr), and T cells. We used the published experimental data to set up an ODE model describing the change in the number of immune cell populations over time during DSS colitis. Dynamic changes depend on parameters representing rate constants *k*. First, we tested different model structures by estimating rate constants given the experimental data. We then performed model reduction based on profile likelihood estimation^17^. To this end, we included cell population-specific turnover rates *k*_*turn*_, as a ratio of proliferation to death (see Methods section). For example, the turnover rate *k*_*turnB*_ of B cells is proportional to the accumulation of B cells in the mucosa and indirectly proportional to the depletion of B cells from the colonic tissue (Fig. 1b). DSS is supplied as input to the model. It is present at the given concentration (2.5%) for seven days and absent for the remaining seven days of the experiment (Fig. 1c). The mathematical model included DSS-induced accumulation of tissue damage as well as a parameter representing mucosal healing, *k*_*heal*_. The rates of the ODE model represent first-order mass action kinetics. From the original publication, we concluded, that B cells proliferate in response to mucosal healing^16^. In order to implement into the mathematical model whether DSS treatment or subsequent tissue damage impacts the other three immune populations during murine colitis, we performed model selection based on the corrected Akaike Information Criterion^18^. All eight possible combinations were tested (Supplementary Table 1). Model number 6 was chosen as best model (Supplementary Fig. 1a). The best model included stimulation of neutrophils by DSS, whereas macrophages and T cells were stimulated by tissue damage (*c.f*. Figure 1b). Equations of the best mathematical model are given in the methods section.

During model fitting, a global optimum was found in 91% of the multi-start parameter estimation runs (Supplementary Fig. 1b), indicating robust optimisation^19^. Our fitted model with five dynamic rate constants and four initial concentrations proved to be fully structurally and practically identifiable^20^ (Supplementary Fig. 1c). We obtained a fit with a Pearson’s rho of 0.88 between experimental data and model simulation (Supplementary Fig. 1d).

Our fitted model was able to capture the dynamics of the four immune cell populations from the FACS dataset (Fig. 1d). After a subtle early drop, B cells accumulated over time (with model simulations lagging slightly behind experimental data). Macrophages and T cells showed qualitatively similar behaviour. They both increased until day 10 and then declined hyperbolically. Here, the high data variability at day 10 influences the simulation, especially for macrophages. Neutrophils sharply peaked at day 7 and then declined almost linearly (Fig. 1d).

In summary, longitudinal flow cytometry data seems to be suitable for deterministic modelling of immune cell population dynamics during murine colitis. The mathematical model further supports the notion that B cells expand during mucosal healing^16^, which is preceded by neutrophils peaking with tissue damage.

### Independent validation of the best ODE model with single-cell data

**Fig. 2:**
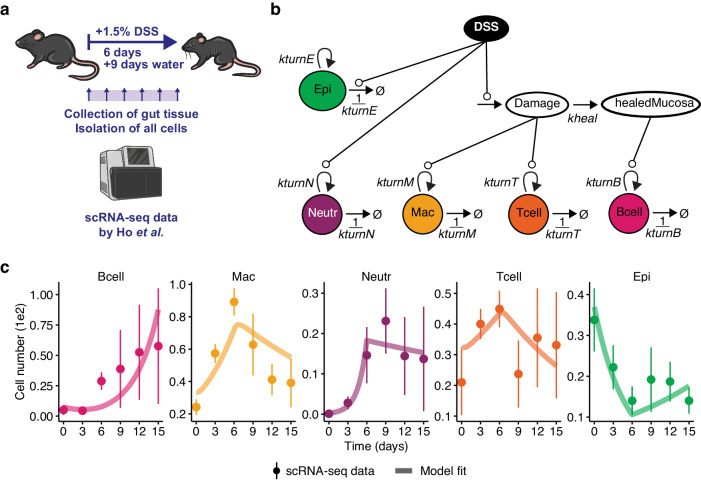
Schemes and model fit for scRNA-seq data. **a** Scheme of the single-cell mRNA-sequencing (scRNA-seq) experiment published by Ho et al. **b** Schematic representation of the extended mathematical model. **c** Experimental scRNA-seq data and simulations of the fitted model. Points represent mean values; error bars indicate standard deviations from ten technical replicates per time point. Solid lines represent model simulations of the best fit.

To see whether our selected best mathematical model generalizes to a different experimental protocol of DSS colitis, a different single-cell technology, and thus a different type of dataset, we sought to independently validate the model structure (*c.f*. Figure 1b). To this end, we obtained publicly available longitudinal scRNA-seq data from Ho et al. (GSE148794) ref. ^21^. In this protocol, mice received 1.5% DSS in the drinking water for six days and afterward water for another nine days. Importantly, data was collected longitudinally during the experiment, meaning that mice were sacrificed and colon samples were collected at days 0, 3, 6, 9, 12, and 15 (Fig. 2a). Per time point, three biological replicates (mice) were pooled and subjected to scRNA-seq by Smart-Seq2 ref. ^22^. The mouse information was not hashed, but ten technical replicates were sequenced per time point, allowing us to estimate the uncertainty in the dynamics of cell numbers. To identify corresponding cell populations from the flow cytometry dataset in the scRNA-seq dataset, we reanalyzed the data from Ho et al.^21^. Upon filtering and unsupervised clustering, we obtained 14,606 quality-control-positive cells in 20 clusters (Supplementary Fig. 2a). As the dataset also contained non-immune cells, we decided to expand our analysis to epithelial cells, which are highly relevant to IBD pathophysiology^23^. We were able to discriminate between immune and epithelial cells by their respective expression of the canonical markers *Ptprc* (CD45) and *Epcam* in the scRNA-seq dataset (Supplementary Fig. 2b). To annotate corresponding clusters of B cells, macrophages, neutrophils, and T cells, we also inspected *Fcgr1* (CD64), *Itgam* (CD11b), *Itgax* (CD11c) and *Thy1* (CD90), which were all used as surface markers in the flow cytometry data by Frede et al.^16^ (Supplementary Fig. 2c). Neutrophil annotation was corroborated by their expression of S100a8/9 ref. ^21^. We then extracted cell numbers of the five annotated cell type clusters shown in Supplementary Fig. 2d, assuming that they were evenly sampled from pooled specimens. Epithelial turnover was implemented in the mathematical model depending on DSS concentration (Fig. 2b), which was simulated as model input (Supplementary Fig. 2e). When fit to the scRNA-seq data, the extended mathematical model with six dynamic rate constants and five initial concentrations proved to be fully structurally and practically identifiable (Supplementary Fig. 2f). We obtained a fit with a Pearson’s rho of 0.86 between experimental data and model simulation (Supplementary Fig. 2g). Our fitted model was able to capture the dynamics of the cell numbers extracted from the reanalysed scRNA-seq dataset (Fig. 2c). As in the flow cytometry data (*c.f*. Figure 1d), B cells accumulated over time (with model simulations lagging slightly behind experimental data). Macrophages and T cells showed qualitatively similar behaviour. They both increased until day 6 and then declined almost linearly. Neutrophils also peaked at day 6 and then slightly descended, while epithelial cells declined until day 6 and then recovered (Fig. 2c).

It should be noted that we used the same core mathematical model with two completely independent studies, obtained by different technologies; flow cytometry vs. scRNA-seq. The experimental protocol, i.e., DSS concentration and timing, was different, which we simulated as input accordingly. In general, there are several statistical models to infer robust changes in cell numbers from scRNA-seq data, e.g., scCODA^24^, or sccomp^25^. A continuous model for testing for differential abundance in scRNA-seq data is Milo^26^. Of note, none of these models are designed for time series data.

In summary, our selected deterministic model captures cell type dynamics during different murine DSS colitis protocols, highlighting overarching mechanisms of B cells expanding during mucosal healing and neutrophils peaking early during DSS-induced tissue damage.

### Predictive power of an ODE model fitted to longitudinal scRNA-seq data

We then asked whether the estimated rate constants of the fitted ODE model were indicative of cellular gene expression. If this is the case, transcriptional programmes inferred from scRNA-seq data can potentially inform parameter estimation, e.g. by determining priors based on expression levels. For example, if the expression of a cell cycle score of a cell population is rather high in the scRNA-seq data, one would expect to find a high estimate of the rate constant for the proliferation parameter of the cell population in the ODE model. To test this hypothesis, we examined the confidence bounds of the optimised turnover rates per cell type (Supplementary Fig. 3a). These estimated parameters were used as model predictions. High turnover rates reflect the expansion of cells in the mucosa or *lamina propria* during DSS colitis (as a change in cell number per day). Immune cells with high turnover rates are therefore expected to be more pro-inflammatory, while non-immune cells with high turnover rates would imply mucosal healing under these conditions. We reasoned that a gene expression signature reflecting both intestinal inflammation and tissue damage would best correlate with the model prediction of estimated turnover rates. We selected the gene module of the KEGG pathway hsa05321: ‘Inflammatory bowel disease – Homo sapiens (human)’ for experimental validation. After converting the genes to their murine homologues, we calculated the IBD expression signature from the scRNA-seq data as experimental validation (Supplementary Fig. 3b). Note that gene expression was not used for model fitting. The estimated turnover rates and the mean of the calculated IBD expression score correlated with Pearson’s rho=0.92 (Supplementary Fig. 3c). Model predictions of turnover rates were thus independently validated by expression of the IBD gene module.

**Fig. 3:**
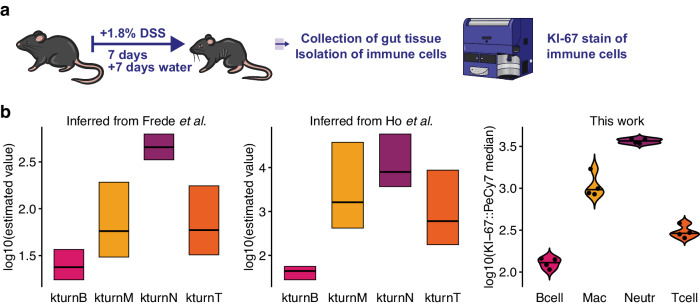
KI-67 validation. **a** Scheme of the KI-67 staining experiment of this work. **b** 95% confidence intervals of estimated immune cell turnover rates fit the data by Frede et al., or the data by Ho et al., and corresponding KI-67 staining of this work. A horizontal line within the confidence bounds of the parameters indicates the global optimum. Violin plots of median KI-67 protein level of immune cell populations measured by flow cytometry. Every dot represents a mouse.

As the IBD gene module contains 65 genes, we wondered whether a simpler physiological molecular readout would also reflect the estimated turnover rates. We turned to KI-67, which serves as a proliferation marker^27^. To test our hypothesis that estimated rate constants represent biological processes, we performed a dedicated experiment. We treated mice with 1.8% DSS as an effective concentration for seven days and allowed them to recover with water for a further seven days (Fig. 3a). With our DSS dose, mice lost weight during the phase of inflammation and recovered during mucosal healing (Supplementary Fig. 3d). We then isolated colonic immune cells and stained them for the antigen KI-67 (Supplementary Fig. 3e, f). Our analyses show that the estimated turnover rates of the immune cell populations are coherent between our two model setups, and indicative of KI-67 protein levels measured by median fluorescence intensity (Fig. 3b). High levels of KI-67 (and proliferation) in macrophages were reported earlier, since they phagocytose apoptotic cells to resolve tissue injury^28^. Short-lived neutrophils do not proliferate in the periphery and rather show high turnover due to rapid recruitment and subsequent depletion from the tissue^29^. High levels of KI-67 in neutrophils have been linked to their functional properties, such as neutrophil extracellular trap (NET) formation^30^.

Taken together, our results indicate that estimated turnover rates can be informed by gene expression data and at the same time be predictive of biological processes.

### Translational implications of an ODE model of murine colitis

**Fig. 4:**
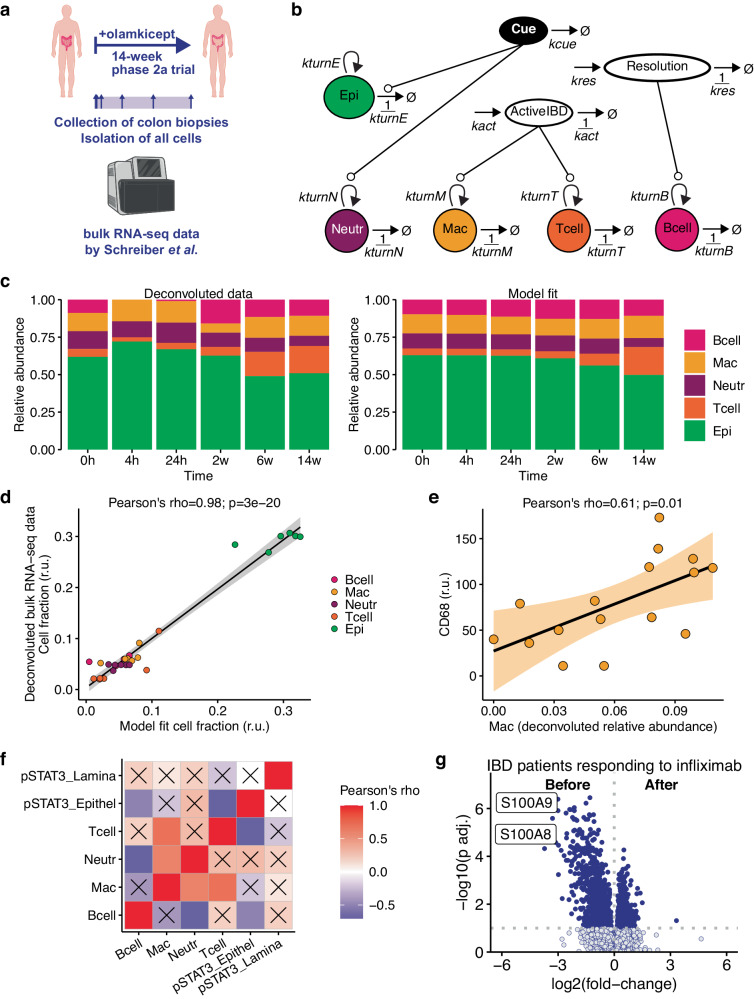
Schemes, model fit and clinical relevance. **a** Scheme of the open-label phase 2a clinical trial published by Schreiber et al. IBD patients with active disease who went in remission at week 14 after initial treatment were selected. **b** Schematic representation of the mathematical model. **c** Deconvoluted bulk RNA-seq data. Inferred relative abundance of cell types in colon biopsies of IBD patients over time after initial olamkicept treatment and model fit. **d** Correlation between relative cell fractions obtained by deconvolution of bulk RNA-seq data and model fit. Shaded region indicates the 95% confidence interval of the linear fit. **e** CD68 measured by multiplexed immunohistochemistry in colon biopsies of IBD patients (measured by Schreiber et al.) vs. deconvoluted relative abundance of macrophages. Shaded region indicates the 95% confidence interval of the linear fit. **f** Cross-correlation between STAT3 phosphorylation in epithelial region or *lamina propria* of colon biopsies of IBD patients and deconvoluted relative abundance of immune cells. Crosses indicate a significant correlation *p* < 0.05. **g** Vulcano plot of differentially expressed genes in colonic biopsies from 27 IBD patients responding to anti-TNF therapy. Data from Arjis et al. Before vs. after initial treatment. Mann-Whitney U test. *p* values adjusted for multiple testing.

Finally, we investigated whether our extended ODE model reflected processes in human IBD. To this end, we looked for publicly available longitudinal data from clinical trials. We found bulk mRNA-sequencing (bulk RNA-seq) data of colon mucosal biopsies from IBD patients enrolled in an open-label, prospective phase 2a trial, deposited as GSE171770 by Schreiber et al.^31^ In this study, 16 IBD patients with active disease, all of which experienced failure with conventional therapies, received the interleukin (IL-)6 trans-signalling inhibitor olamkicept. During the course of the 12-week treatment, colon biopsies were taken at 0 h, 4 h, 24 h, 2 weeks, 6 weeks, and 14 weeks after initial treatment and subjected to bulk RNA-seq (Fig. 4a). Clinical effectiveness of IL-6 inhibition was corroborated by the analysis of downstream targets, in this case: phosphorylation of STAT3. Based on clinical parameters such as disease activity scores, three out of the 16 patients were in remission. We focused our analysis on these individuals (two UC, one CD) in remission, as it is the clinical setting closest to a resolution of inflammation and mucosal healing in the murine DSS colitis protocol.

Before subjecting the longitudinal information to our mathematical model, we had to deconvolute the bulk RNA-seq data (see methods). First, we tried all cell types discovered by scRNA-seq from human UC patients by Smillie et al.^32^ Due to dominating expression patterns, among the 51 queried cell types, only 21 were detected in the data from Schreiber et al., mostly assigned to cycling B cells or plasma cells (Supplementary Fig. 4a). We therefore decided to use the cell types of our analysis from Ho et al. as reference for the deconvolution of the human bulk RNA-seq data from Schreiber et al. We detected B cells, epithelial cells, macrophages, neutrophils and T cells in the human data while substantial fractions were also assigned to ‘Other’ cells as expected (Supplementary Fig. 4b). Then, we had to adapt the structure of the mathematical model because the trial (and thus sampling) started in patients with active disease rather than at the onset of the disease (as in the murine DSS colitis protocol). The setup for all cell types remained the same as in the extended mathematical model of the murine DSS colitis (Fig. 4b). But the DSS input was replaced by a ‘Cue’ that stimulated neutrophils and led to epithelial decay. ‘Damage’ was replaced an ongoing ‘ActiveIBD’, which stimulated macrophages and T cells as in the mathematical model of the murine DSS colitis. The healing mucosa was modelled as an independent variable ‘Resolution’, which in turn led to B cell expansion consistent with the extended mathematical model.

The deconvoluted data and the model fit indicated a relative expansion of T cells and a depletion of neutrophils in colon biopsies of IBD patients in remission 14 weeks after initial olamkicept treatment (Fig. 4c). We obtained a fit with a Pearson’s rho of 0.98 between deconvoluted data and model simulation (Fig. 4d). To further corroborate the reliability of the deconvoluted cell fractions, we performed correlation analysis comparing relative abundance of cell types with canonical markers of immune cells measured by multiplexed immunohistochemistry from the same colon biopsies that were also used for bulk RNA-seq^31^. We found a significant positive correlation between CD68 and the deconvoluted relative abundance of macrophages (Fig. 4e). Also the correlation between measured myeloperoxidase (MPO) and deconvoluted relative abundance of neutrophils was positive, but not significant (Supplementary Fig. 4c). A negative correlation, although not significant, was found between CD3 and deconvoluted relative abundance of T cells (Supplementary Fig. 4d), which points at an overestimation of T cell abundance. This limitation of deconvolution can occur if reference cell types depend on lowly-expressed markers, which are not detected, and therefore cells are incorrectly assigned. In the original study by Schreiber et al.^31^, the authors also concluded that neutrophils are depleted from the colon of IBD patients in remission, even though they found no significant differences in MPO levels between patients in remission and those who did not go in remission. Through the deconvolution, we found additional evidence that neutrophil depletion is clinically relevant to IBD remission, namely the neutrophil decline in the model fit (*c.f*. Figure 4c), and the positive correlation with MPO (*c.f*. Supplementary Fig. 4c). In addition, we found that deconvoluted relative abundance of neutrophils most strongly correlated with measured pSTAT3 in the colon epithelium and *lamina propria* of IBD patients in remission (Fig. 4f). This observation suggests that neutrophil depletion can indicate whether inhibition of IL-6 downstream signalling through olamkicept worked in IBD patients in remission. Neutrophil counts were also included as secondary end point in a follow-up randomized control trial with olamkicept in IBD patients^33^.

As our analysis results are only based on three IBD patients in remission and neutrophils are hard to detect by scRNA-seq^34^, we were looking for additional evidence supporting our hypothesis that neutrophil depletion from the colon is indicative of an ongoing IBD therapy response and/or remission. We obtained publicly available Affymetrix microarray data from Arijs et al. (GSE16879) ref. ^35^ from 61 IBD patients before and four to six weeks after their first infliximab infusion, which represents an anti-TNF therapy. We looked for differential gene expression in the colon biopsies of 27 responders before and after initial treatment. In this unsupervised analysis we found the neutrophil marker genes *S100A8* and *S100A9* among the top differentially expressed genes, which are down-regulated upon successful response to therapy (Fig. 4g). While this observation might be a mere indication that the inflammation in the tissue starts being resolved, it adds to our understanding of IBD as a pathophysiological process.

In conclusion, our ODE model allows the simulation of colon cell dynamics in IBD patients and indicates a potential role of neutrophil depletion for IBD patients to respond to treatment and to enter remission.

## Discussion

In this study, we explored the extent to which longitudinal single-cell-based data can inform deterministic modelling of murine colitis. The amount of publicly available omics datasets is steadily increasing, and we expect more longitudinal studies to be published in the future. Here, we provide a proof-of-concept study showing that flow cytometry as well as Smart-Seq2 data can be used to infer cell population abundance dynamics. Although there is still considerable technical and/or biological variability (Fig. 1d, Fig. 2c), the extracted information is sufficient to identify a set of kinetic parameters and initial values of an ODE model describing the change in colonic cell population abundance during murine colitis. We originally developed ‘Data2Dynamics'^36^, an open-source framework for deterministic modelling, but it only runs under the proprietary software MATLAB. Meanwhile, free and open R packages ‘dMod’ and ‘cODE’ are available with similar functionality. With this setup, we were able to capture the extracted dynamics from Frede et al. and Ho et al. To corroborate the estimated immune cell type-specific turnover rates of the fitted ODE model, we performed an independent flow cytometry measurement of murine DSS colitis (Fig. 3a). As flow cytometry allows longitudinal single-cell-based measurements, we have recently used such data for deterministic modelling of erythroid fate decisions^37^. We further validated the estimated turnover rates of our fitted ODE model by comparing them with single-cell gene expression levels of an IBD gene module (Supplementary Fig. 3a, b) and KI-67 staining (Fig. 3b). Future research will show whether differentiation markers or other reporters are also suitable to provide information on turnover rates in murine colitis models that not only address net proliferation or activation but also apoptosis^3,38^.

Studies on human IBD increasingly focus on transcriptomic readouts^39–43^. One of the major challenges will be the implementation of these data into mathematical models for robust inference of biological properties. We envision that deconvolution of time-resolved bulk RNA-seq data, as we did in Fig. 4c, could complement scRNA-seq resources, e.g., from the Human Cell Atlas^44^, for ODE-based modelling to leverage large-scale single-cell data in clinical practice. We have previously shown that multiple colon biopsies from the same site of an individual ulcerative colitis patient yield similar cell type frequencies when subjected to scRNA-seq^32^. However, high inter-individual variability, cell type robustness, isolation protocols, and technologies are major sources of measurement error. The former could be addressed with more sophisticated statistical models such as mixed-effect models of cell type-specific pseudo bulks^45^. Another limitation in inferring cell abundance dynamics from scRNA-seq data with clinical relevance is the lack of spatial resolution. The sensitivity of the technologies is steadily improving, so that partial differential equation models will soon be able to infer cellular and molecular dynamics across space and time for systems immunology in IBD and beyond.

## Methods

### Mice

All mice were of C57/BL6J wild-type background and bred in the UKE animal facility. Animals were kept in accordance with the institutional review board ‘Behörde für Soziales, Familie, Gesundheit und Verbraucherschutz’ (Hamburg, Germany), and under specific-pathogen-free conditions at ambient temperature. Experimental procedures were approved by the local ethics committee under the number N033/23. Mice were all male and between 8 and 12 weeks of age.

### DSS-induced murine colitis model

Murine colitis was induced by administration of 1.8% DSS (Alfa Aesar) as the effective concentration dissolved in drinking water *ad libitum* for seven days, followed by seven days of pure water. Mouse physiology and body weight were monitored regularly. After a total of 14 days, the mice were sacrificed by cervical dislocation upon anaesthesia with a CO_2_/O_2_ mixture at 80%/20% ratio, and the colons were collected for further processing.

### Isolation of colonic cells

Excised colon samples were extensively washed of luminal content and opened longitudinally. The tissue was cut into 1 cm pieces and then incubated in HBSS (Gibco) containing 5 mM EDTA, 5% FBS, 10 mM HEPES and 1 mM DTT at 37 °C with shaking for 20 min. Samples were then digested in HBSS with CaMg, 1 mg/ml collagenase VIII (Sigma), 0.4 mg/ml DNAase I, 5% FBS and 10 mM HEPES at 37 °C using the gentleMACS Octo Dissociator with Heaters’ programme ‘37C_m_LPDK_1’. Isolated cells were washed once in PBS (2 mM EDTA, 1% FBS) and lymphocytes were enriched on a 40–67% Percoll gradient (GE Healthcare).

### Flow cytometry

Cells were stained with live/dead dye and antibodies of the following mixture, which was from BioLegend and 1:200 dilution, unless stated otherwise: CD45 (clone: 30-F11, BD Biosciences Cat# 565967), CD90.2 (clone: 53-2.1, Cat# 140317), CD3 (clone: 17A2, Cat# 100204, 1:400), B220 (clone: RA3-6B2, Cat# 103243), I-A/I-E (clone: M5/114.15.2, Cat# 107631, 1:400), CD11b (clone: M1/70, Cat# 101207, 1:800), CD64 (clone: X54-5/7.1, Cat# 139311, 1:300) and, Ly-6G (clone: 1A8, Cat# 127609, 1:600) surface antibodies at 4 °C for 20 min. After washing twice, intracellular staining for KI67 (clone: B56, BD Biosciences Cat# 561283) was performed on fixed cells using the Foxp3/Transcription Factor Staining Buffer Set (eBioscience) according to the manufacturer’s instructions. Multiparameter analysis was performed on an LSR Fortessa II (BD) and analysed with FlowJo software (FlowJo, LLC).

### mRNA-sequencing analysis

scRNA-seq data was processed with the R package ‘Seurat’ (v. 5.0.3)^46^.

For re-clustering of the data from Ho et al., cells with more than 250 but less than 6000 features were selected. Data was log-normalized, and the top 2000 most variable features were selected by the ‘vst’ method. Clusters were determined based on the top 20 principal components with a ‘resolution’ = 0.5.

Convolution of scRNA-seq data to pseudo-bulk information was performed with the ‘AggregateExpression’ function. The deconvolution was performed with the R package ‘DeconRNASeq’ (v. 1.40.0)^47^. Such analysis of ‘mixed’ populations yields relative fractions of cell types per bulk RNA-seq sample based on a ‘pure’ reference dataset per cell type. To generate such a reference dataset from the scRNA-seq data from Ho et al., pseudo-bulk expression patterns were generated for all cell types. Those murine gene expression patterns were then converted from murine to human homologues using the R package ‘nichenetr’ (v. 1.1.1)^48^.

Affymetrix microarray analysis was performed with the R packages ‘Biobase’ (v. 2.58.0) and ‘GEOquery’ (v. 2.66.0). Differential gene expression analysis of Affymetrix microarray data was performed by Mann-Whitney U test with false-discovery rate (FDR) correction.

### Statistical analysis

Unless stated otherwise, group comparisons were performed by non-parametric Mann-Whitney U test, with correction for multiple testing (when applicable).

### Deterministic modelling

Our deterministic model assumes that the change of cell number of populations $\vec{C}$ with time $t$ during colitis can be described as a function of those cell populations $\vec{C}$ and estimated parameters $\vec{k}$:

1 $$\frac{d\vec{{{C}}}}{d{{t}}}={{f}}\left(\vec{{{C}}},\vec{{{k}}}\right).$$

Each cell type-specific turnover rate ${k}_{{turn}}$ is modelled as the ratio between proliferation rate ${k}_{{prol}}$ and death rate ${k}_{{death}}$:

2 $${k}_{{turn}}=\frac{{k}_{{prol}}}{{k}_{{death}}}$$

ODEs in the core model of the murine system are given as follows:

3 $$\frac{d{Damage}}{dt}=+{DSS}-{k}_{h{eal}}\times {Damage}$$

4 $$\frac{{dh}{ealedMucosa}}{dt}=+{k}_{h{eal}}\times {Damage}$$

5 $$\frac{{dBcell}}{{dt}}=\left(+{k}_{{turnB}}\times {healedMucosa}-\frac{1}{{k}_{{turnB}}}\right)\times {Bcell}$$

6 $$\frac{{dMac}}{{dt}}=\left(+{k}_{{turnM}}\times {Damage}-\frac{1}{{k}_{{turnM}}}\right)\times {Mac}$$

7 $$\frac{{dNeutr}}{{dt}}=\left(+{k}_{{turnN}}\times {DSS}-\frac{1}{{k}_{{turnN}}}\right)\times {Neutr}$$

8 $$\frac{{dTcell}}{{dt}}=\left(+{k}_{{turnT}}\times {Damage}-\frac{1}{{k}_{{turnT}}}\right)\times {Tcell}$$

With an additional ODE in the extended model of the murine system:

9 $$\frac{d{Epi}}{dt}=\left(+{k}_{{turnE}}-\frac{1}{{k}_{{turnE}}}\times {DSS}\right)\times {Epi}$$

The ODE model of the human system is given as follows:

10 $$\frac{d{Cue}}{dt}=-{k}_{{cue}}\times {Cue}$$

11 $$\frac{d{ActiveIBD}}{dt}=+{k}_{{act}}-\frac{1}{{k}_{{act}}}\times {ActiveIBD}$$

12 $$\frac{d{Resolution}}{dt}=+{k}_{{res}}-\frac{1}{{k}_{{res}}}\times {Resolution}$$

13 $$\frac{d{Bcell}}{dt}=\left(+{k}_{{turnB}}\times {Resolution}-\frac{1}{{k}_{{turnB}}}\right)\times {Bcell}$$

14 $$\frac{d{Mac}}{dt}=\left(+{k}_{{turnM}}\times {ActiveIBD}-\frac{1}{{k}_{{turnM}}}\right)\times {Mac}$$

15 $$\frac{d{Neutr}}{dt}=\left(+{k}_{{turnN}}\times {Cue}-\frac{1}{{k}_{{turnN}}}\right)\times {Neutr}$$

16 $$\frac{d{Tcell}}{dt}=\left(+{k}_{{turnT}}\times {ActiveIBD}-\frac{1}{{k}_{{turnT}}}\right)\times {Tcell}$$

17 $$\frac{d{Epi}}{dt}=\left(+{k}_{{turnE}}-\frac{1}{{k}_{{turnE}}}\times {Cue}\right)\times {Epi}$$

The ODE model was implemented with R packages ‘dMod’ (v. 1.0.2) and ‘cOde’ (v. 1.1.1), which are available at https://github.com/dkaschek/. Default settings were used for optimization runs, with *normL2* as objective function and priors with *mean* = 1e0, *sigma* = 1e1. 100 fits were run per optimization with *mstrust* optimizer. The objective function is the weighted sum of squared residuals:

18 $${\chi }^{2}\left(k\right)=\mathop{\sum }\limits_{i=1}^{m}\mathop{\sum }\limits_{j=1}^{d}{\left(\frac{{y}_{{ij}}^{D}-{y}_{i}(k,{t}_{j})}{{\sigma }_{{ij}}^{D}}\right)}^{2}$$

where ${y}_{{ij}}^{D}$ denotes $d$ data points for all observables $i$ at timepoint ${t}_{j}$. ${\sigma }_{{ij}}^{D}$ refers to the respective measurement error, and ${y}_{i}(k,{t}_{j})$ is the simulation of the observable $i$ at timepoint ${t}_{j}$ as a function of the parameters $k$. Parameter estimation was performed by minimizing the objective function:

19 $${\hat{k}}={\rm{arg}}\,{\rm{min}} \left[{\chi }^{2}\left(k\right)\right].$$

For normally distributed measurement error, this corresponds to the maximum likelihood estimate of $k$, in turn:

20 $${\chi }^{2}\left(k\right)={const}-2\,\cdot\, \log (L\left(k\right))$$

with $L\left(k\right)$ represents the likelihood.

### Supplementary information

Supplementary Information

## Data Availability

All code and data required to reproduce figures is freely available at: https://github.com/AdlungLab/scIBDmod.
