# Supplementary material for: Longitudinal single-cell data informs deterministic modelling of inflammatory bowel disease
Source: NPJ Syst Biol Appl. 2024 Jun 24;10:69. doi: 10.1038/s41540-024-00395-9 (PMC11196733; doi:10.1038/s41540-024-00395-9)
Supplement: Supplementary file 1 — Supplementary Information [file 41540_2024_395_MOESM1_ESM.pdf]

***Supplementary Table 1***
**Model selection**

| i | bestFit | n | k | DSS | Damage | AIC | cAIC | Identifiable at CI [%] |
|---|---|---|---|---|---|---|---|---|
| 1 | 40.7 | 20 | 9 | Mac, Neutr, Tcell | | 76.7 | 94.7 | 68 |
| 2 | 33.7 | 20 | 9 | Mac, Neutr | Tcell | 69.7 | 87.7 | 95 |
| 3 | 44.0 | 20 | 9 | Mac, Tcell | Neutr | 80.0 | 98.0 | 90 |
| 4 | 28.7 | 20 | 9 | Neutr, Tcell | Mac | 64.7 | 82.7 | 95 |
| 5 | 39.5 | 20 | 9 | Mac | Neutr, Tcell | 75.5 | 93.5 | 95 |
| 6 | 20.1 | 20 | 9 | Neutr | Mac, Tcell | 56.1 | 74.1 | 95 |
| 7 | 32.5 | 20 | 9 | Tcell | Mac, Neutr | 68.5 | 86.5 | 95 |
| 8 | 28.6 | 20 | 9 | | Mac, Neutr, Tcell | 64.6 | 82.6 | 95 |

i = Model index, bestFit = objective function, n = number of data points, k = number of parameters, DSS indicates DSS-dependent cell populations, Damage indicates damage-dependent cell populations, AIC = Akaike Information Criterion, cAIC = corrected Akaike Information Criterion, CI = Confidence Interval.

# Supplementary Figure 1

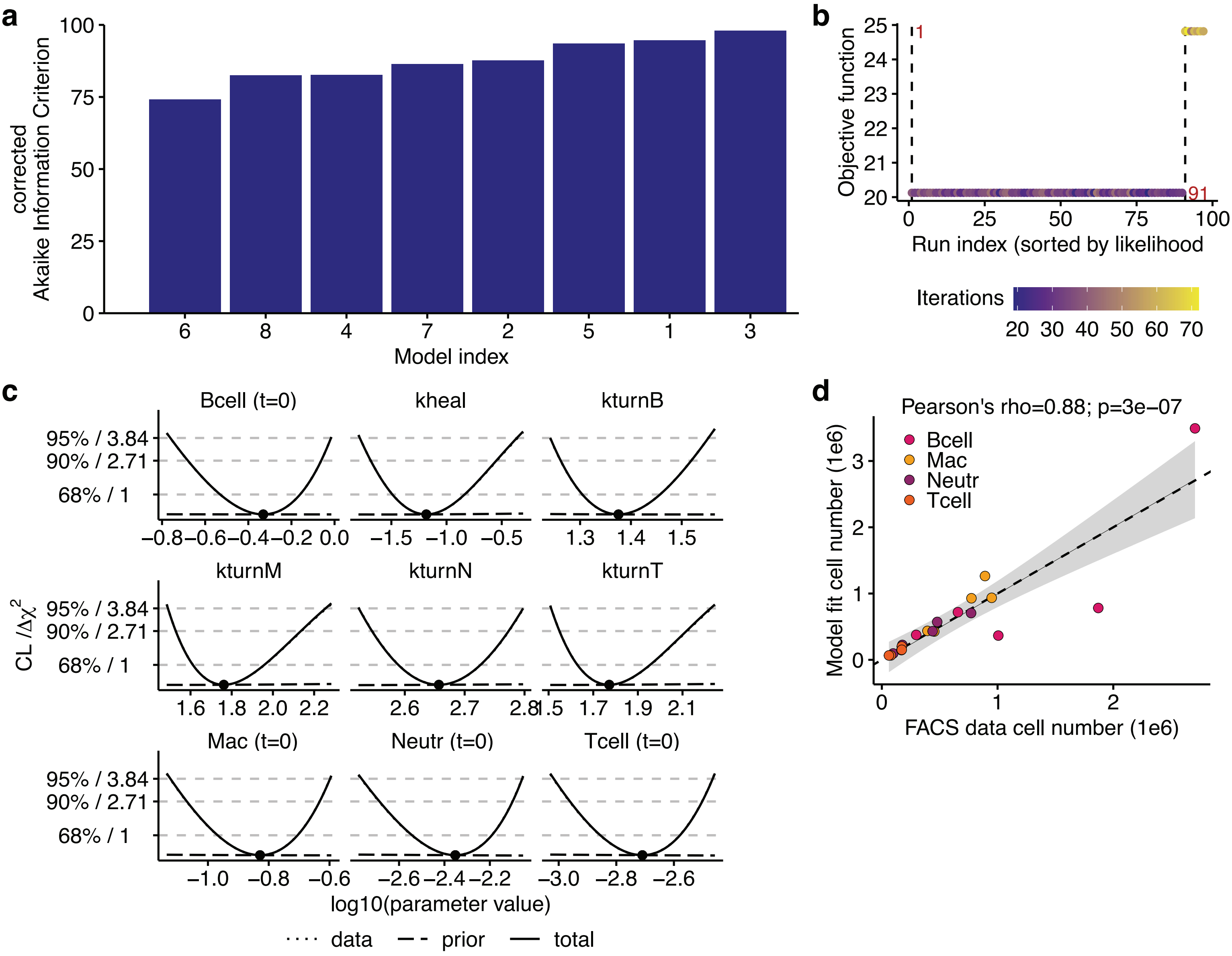

***Supplementary Figure 1***

**Model selection, identifiability and fit.**

**a** Indices of different model structures fitted to experimental data and evaluated by the corrected Akaike Information Criterion. Model 6 was selected as best model.

**b** Multi-start parameter estimation runs with likelihood as objective function.

**c** Profile likelihood estimates of the parameters of the selected best model.

**d** Correlation between cell numbers obtained from FACS data by Frede *et al*. and model fit. Shaded region indicates the 95% confidence interval of the linear fit.

# Supplementary Figure 2

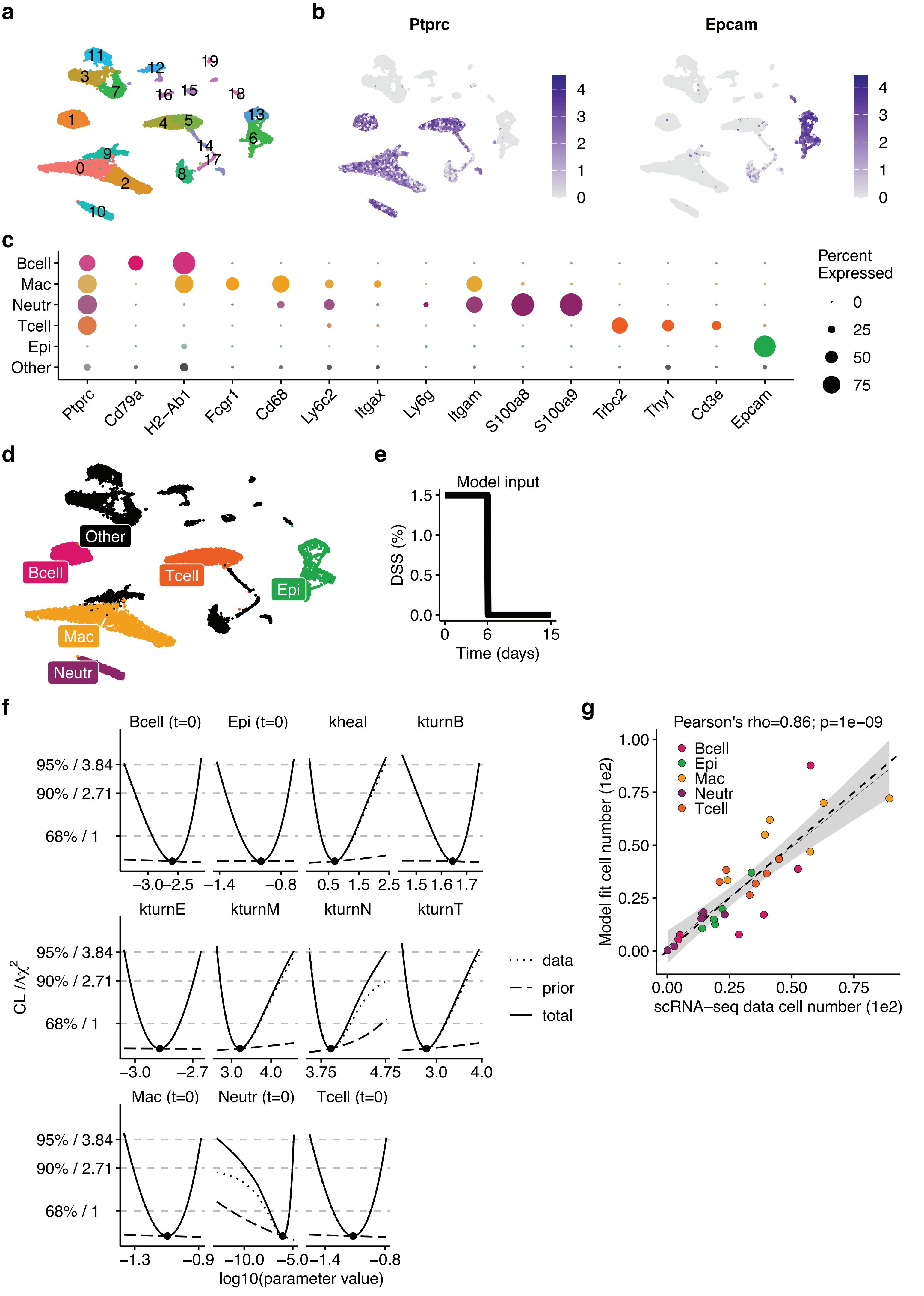

## *Supplementary Figure 2*

**Re-clustering, marker gene expression, model identifiability and fit.**

**a** Re-clustering of UMAP of scRNA-seq data from Ho *et al*. Total of 14,606 quality-control positive cells allocated to 20 clusters.

**b** Scaled gene expression of *Ptprc* (CD45) and *Epcam*.

**c** Bubble plot of selected marker genes.

**d** UMAP of annotated cell types.

**e** DSS simulated as model input.

**f** Profile likelihood estimates of the parameters of the selected best model.

**g** Correlation between cell numbers obtained from scRNA-seq data by Ho *et al*. and model fit. Shaded region indicates the 95% confidence interval of the linear fit.

# Supplementary Figure 3

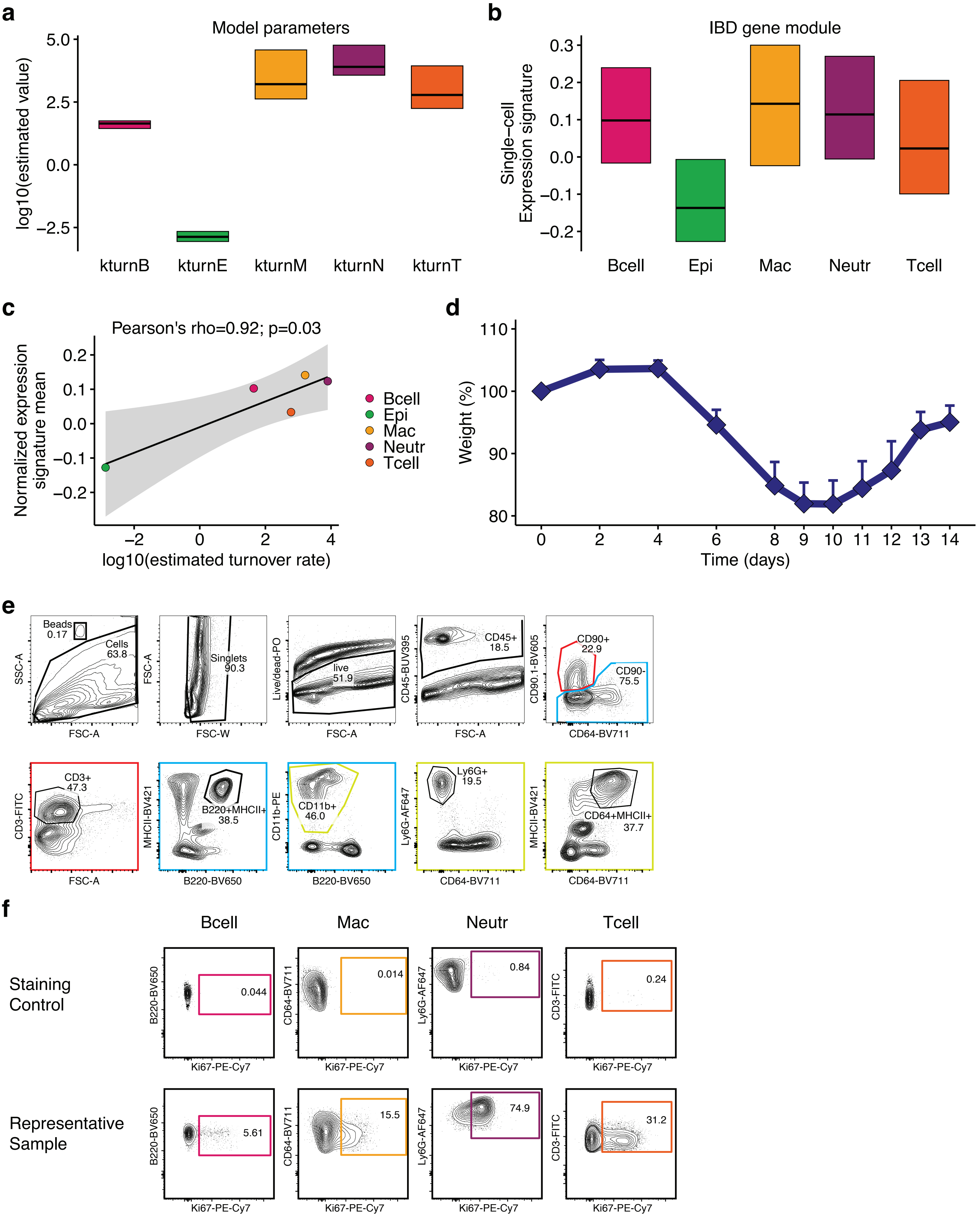

***Supplementary Figure 3***
**Validations and quality controls.**
**a** 95% confidence intervals of estimated cell turnover rates fit to the data by Ho *et al*. Horizontal line within the confidence bounds of the parameters indicates global optimum.
**b** Box plot of normalized gene expression score of IBD gene module (hsa05321) per single cell. Total of 8,899 cells. Upper line indicates 75% percentile, middle line indicates median, lower line indicates 25% percentile.
**c** Correlation between mean values of normalized IBD gene module score per cell type and corresponding best fit values of estimated turnover parameters of the respective cell type. Shaded region indicates the 95% confidence interval of the linear fit.
**d** Relative weight change in DSS experiment, mice were treated with effective concentration of 1.8% DSS in the drinking water for 7 days, and with regular water for another 7 days. Symbols represent mean values of N=4 mice and error bars indicate standard error of the mean.
**e** Gating scheme of colonic immune cells isolated at day 14 of the DSS experiment.
**f** KI-67 staining examples of immune populations. Upper row: Fluorescence Minus One (FMO) control. Lower row: Representative sample.

# Supplementary Figure 4

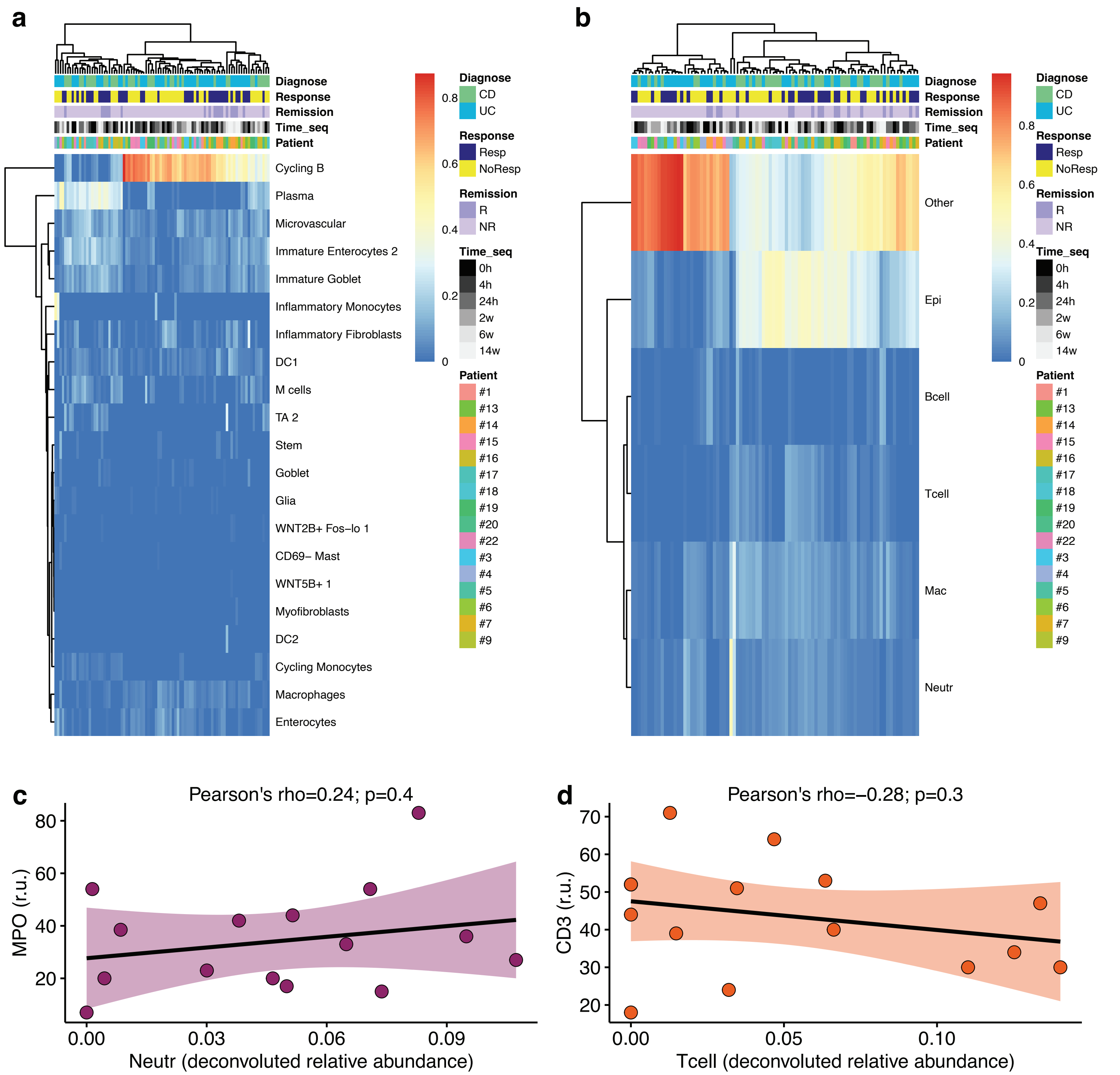

***Supplementary Figure 4***

**Deconvolution and correlation**

**a** Heatmap of deconvoluted bulk RNA-seq data from Schreiber *et al*. with data from Smillie *et al*. as reference.

**b** Heatmap of deconvoluted bulk RNA-seq data from Schreiber *et al*. with data from Ho *et al*. as reference.

**c** Myeloperoxidase (MPO) measured by multiplexed immunohistochemistry in colon biopsies of IBD patients (measured by Schreiber *et al*.) vs. deconvoluted relative abundance of neutrophils. Shaded region indicates the 95% confidence interval of the linear fit.

**d** CD3 measured by multiplexed immunohistochemistry in colon biopsies of IBD patients (measured by Schreiber *et al*.) vs. deconvoluted relative abundance of T cells. Shaded region indicates the 95% confidence interval of the linear fit.
